# Predicting the settlement of coarse granular materials under vertical loading

**DOI:** 10.1038/srep05707

**Published:** 2014-07-16

**Authors:** Juan Carlos Quezada, Gilles Saussine, Pierre Breul, Farhang Radjaï

**Affiliations:** 1 SNCF PSIG, 6 Avenue François Mitterrand, 93574 La Plaine Saint Denis Cedex, France; 2 SNCF Innovation and Research, 40 Avenue des Terroirs de France, F-75611 Paris Cedex 12, France; 3 Institut Pascal, CNRS-Université Clermont II, F-63171 Aubière, France; 4 Université Montpellier 2, CNRS, LMGC, F-34095 Montpellier, France

## Abstract

Granular materials are widely used in industrial processes despite their complex and poorly understood mechanical behaviour both in static and dynamic regimes. A prototypical example is the settlement and compaction of a granular bed under vibrational loading. The elementary mechanisms of this process are still unclear and there is presently no established theory or methodology to predict the settlement and its statistical variability. By means of a parametric study, carried out on a full-scale track, and a critical analysis of density relaxation laws, we introduce a novel settlement model in coarse granular materials under cyclic loading. Our extensive experimental data indicate that the settlement process is governed by three independent parameters strongly correlated with the vibration intensity and initial packing fraction. We show that the mean settlement is well predicted by the model with its parameter values extracted from experimental data.

Granular materials are both pressure-dependent and density-dependent materials and exhibit a broad range of intricate behaviours due to their discrete nature, dissipative interactions and generic structural disorder^1^. The packing fraction may vary as a result of particle rearrangements induced by shearing or vibrations and it leads to dramatic changes in the structure and mechanical response of a granular material^2^,^3^,^4^,^5^,^6^,^7^,^8^. A long-time logarithmic relaxation law of the packing fraction is systematically observed in experiments^9^,^10^. In simple compaction models, this behaviour is attributed to the exponentially increasing time for the particles to reach a new configuration of lower packing fraction. The case of settlement under cyclic loading has, however, been much less investigated. The settlement of granular bed occurs due to both compaction and side-wise spreading. An important industrial example is the railway ballast, which undergoes gradual settlement under the static and dynamic overloads induced by train traffics^11^,^12^,^13^,^14^. The readjustment of differential settlements requires costly operations on fast-train railways. For this reason, an improved understanding of the parameters governing the settlement process is a critical technological challenge for new developments in this field.

In this paper, we show that the total settlement *τ_N_* under vertical cyclic loading is governed by a logarithmic relaxation law as a function of the number N of cycles: 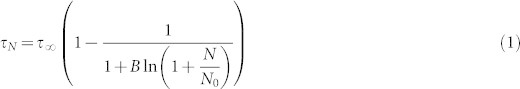
where the three fitting parameters *τ*_∞_, *B* and *N*_0_ can be evaluated from the loading parameters, namely the frequency *ω* (related to the train speed for ballast) and initial packing fraction of the material. Our experimental correlations between model and loading parameters show consistently that *τ*_∞_ and *B* depend on the dimensionless loading intensity Γ = (*Aω*^2^)/(*pd*^2^/*m* + *g*), where *A* is the vibration amplitude, *p* is the confining pressure (under the sleeper for ballast), *d* is the average particle diameter, *m* is the average particle mass and *g* is gravitational acceleration. We also find that the parameter *N*_0_ is linked to the initial packing fraction of the material. In fact, this parameter controls the initial settlement rate, and it was systematically determined by means of a light penetrometer in our experiments on ballast material.

## Results

### Loading system

**Figure 1:**
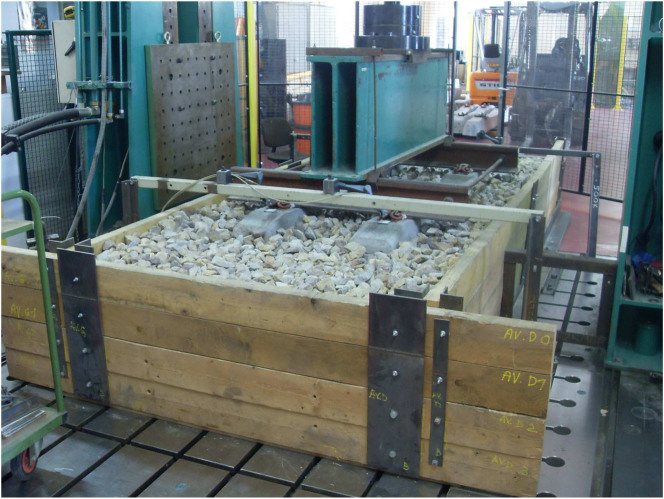
Full-scale track model used in the present work.

We carried out several experimental tests on a full-scale track model composed of a granular bed of ballast particles (Fig. 1). The bed was subjected to a sinusoidally varying vertical overload up to 10^4^ cycles for a broad range of amplitudes and frequencies. In the same way, the initial state of the granular bed was varied and a light dynamic penetrometer was used to characterize its packing fraction before each loading test^15^,^16^,^17^. This device provides the cone penetration strength, which is a parameter strongly dependent on the packing fraction of a granular sample. The principle of the light penetrometer is to push into the material, by manual beating with a hammer, a conic tip of cross-section *A_tip_* = 2 cm^2^. For each blow, the depth of penetration and the input energy are measured to calculate the dynamic cone strength *q_d_* with the corresponding depth using the so-called Dutch formula: 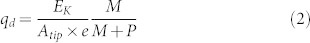
where *M* is the mass of the hammer, *P* is the weight of the driven parts during impact (impact head, road and tip), *E_K_* is the kinetic energy transmitted to the system during the impact and *e* is the penetration per blow. A detailed description of the model and testing apparatus can be found in the Methods section below.

**Figure 2:**
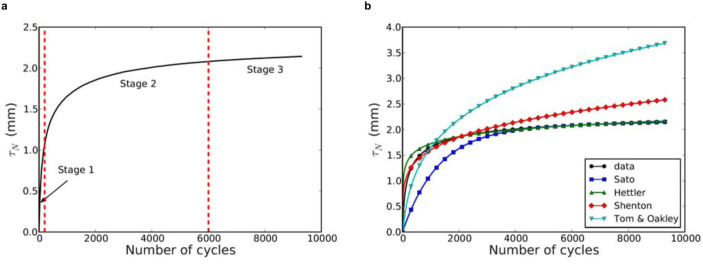
(a) Settlement of ballast material as a function of the number of cycles with its three stages; (b) Comparison between the experimental settlement data obtained from a cyclic loading test with frequency of 3.3 Hz and applied load of 194 × 10^3^N, and several ballast settlement models.

The cumulative settlement may qualitatively be described as occurring in three stages as displayed in Fig. 2a. The first stage is the initial fast compaction of the granular bed during the first 200 cycles. Its quasi-linear evolution reflects the uneven distribution of voids in the material. The largest voids disappear as a result of local particle rearrangements, leading thus to the overall increase of packing fraction and settlement. The second stage lasts from 200 to 6000 cycles with a nonlinear increase of settlement. This “intermediate” stage involves collective particle rearrangements since large voids are exhausted in the first stage. Hence, large-scale particle movements and longer excursions are necessary for increasingly smaller available void space^1^,^9^. This slow relaxation is quite similar to that observed in glasses, spin glasses and flux lattices^18^,^19^,^20^. The last stage of settlement is the long-term behaviour observed beyond 6000 cycles. In this part of the curve, the settlement has a slow linear evolution with the number of cycles. No collective particle rearrangements are observed so that the slow evolution should be attributed to “rare events” such as sudden micro-sliding induced by erosion or grain crushing. Beyond this schematic description, we are interested here in a functional form capable of predicting the settlement for a high number of cycles with parameters physically related to and measurable from packing fraction and loading conditions.

### Best-fit model

We tested different models proposed for the railway ballast settlement based on loading parameters such as vertical stress, subgrade stiffness and calibration constants^21^. None of these models was found to describe satisfactorily our settlement curves shown in Fig. 2b. Shenton expression fits well the beginning of the settlement curve but overestimates long-term settlement. The expressions of Sato and Hettler do not provide a better fit until 5000 cycles. Moreover, a fundamental short-coming of such phenomenological models is that they introduce calibration constants that have no obvious physical interpretation and cannot be estimated by means of independent experimental measurements.

An alternative approach is the density relaxation law for granular materials tested on model systems. This law, called “Chicago fit”, was obtained by experimental analysis of the evolution of packing fraction for mono-disperse spherical particles (glass beads) in a cylindrical tube under a series of external excitations consisting of vertical shakes or “taps” applied to the container^2^,^3^,^9^. This relaxation law is an inverse-logarithmic law: 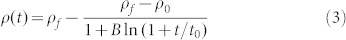
where *ρ_f_* is the ultimate value of packing fraction, *ρ*_0_ is the initial packing fraction, *B* is a fitting parameter and *t*_0_ is a characteristic time. Equation (1) given in Introduction is nothing but a transcription of equation (3) in which the number of cycles *N* replaces time, *τ*_N_ replaces the packing fraction, and the characteristic time is replaced by a characteristic number of cycles *N*_0_. From 360 independent settlement data, the parameters for the two models were identified from the loading parameters (amplitude and frequency) of each test.

**Figure 3:**
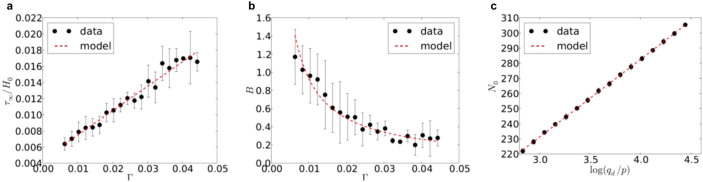
Correlations between the model parameters and loading parameters. (a) The relation between settlement normalized by the thickness of the granular bed *τ*_∞_/*H*_0_ and Γ; (b) The relation between *B* and Γ; (c) The relation between *N*_0_ and *q_d_*/*p*.

**Figure 4:**
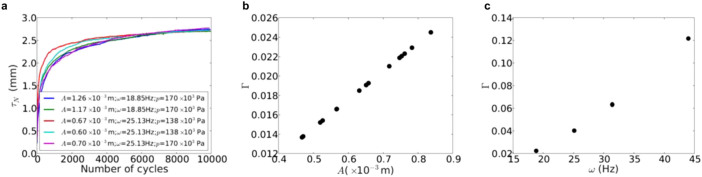
(a) Settlement for different values of *A*, *ω* and *p*, but the same value of Γ; (b) Values of Γ as a function of the amplitude for *ω* = 18.85 Hz and *p* = 138 × 10^3^ Pa; (c) Values of Γ as a function of *ω*, for *A* = 0.8 × 10^−3^ m and *p* = 170 × 10^3^ Pa.

Table 1 shows the inter-correlations coefficients among the model and loading parameters. The parameters *τ*_∞_ and *B* are clearly linked to the dimensionless acceleration Γ whereas *N*_0_ depends on the initial packing fraction of the granular material. The correlation between *τ*_∞_ normalized by the initial thickness of the granular layer *H*_0_ and Γ has a high correlation coefficient *R*^2^ = 0.9 (Fig. 3a), thus suggesting a linear relationship between the two parameters. Different values of amplitude and confining pressure lead to a similar behaviour with the predicted values of *τ*_∞_/*H*_0_. In the same way, for different values of *A*, *ω* and *p* but similar values of Γ, the settlement curves show a quite similar behaviour, as seen in Fig. 4a. The parameters *A*, *ω* are independent from each other, while Γ increase as a function of both (Fig. 4b and 4c). Γ appears thus to be the governing parameter of settlement.

Figure 3b shows the correlation between *B* and Γ. Despite the relative dispersion of data due to the inherent variability of the system, it is seen that *B* globally declines as the acceleration parameter increases. Regarding the *N*_0_ parameter, a logarithmic relationship with the tip strength (normalized by *p*) is observed; see Fig. 3c. Indeed, increasing the initial packing fraction, in correlation with the penetration strength *q_d_*, leads to higher values of *N*_0_ and therefore lower initial slope or slower settlement in the first loading cycles.

**Figure 5:**
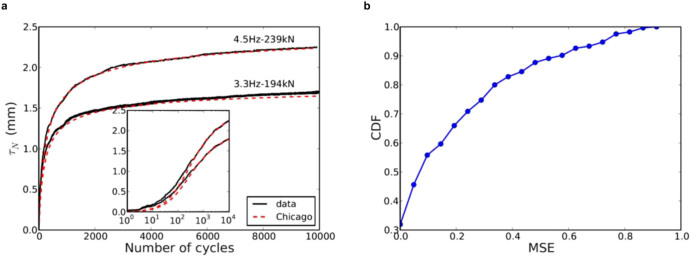
(a) Prediction of settlement by a logarithmic law in comparison to the experimental data for two tests (frequency of 4.5 Hz and applied load of 239 × 10^3^ N; Frequency of 3.3 Hz and applied load of 194 × 10^3^ N). The inset shows the data on a log-linear scale; (b) Cumulative distribution function of the mean square error (MSE) of model prediction compared to 360 experimental tests.

In Fig. 5a the experimental data are compared with the above model of settlement. This model describes quite well the evolution of settlement up to a high number of cycles and for the three stages of settlement when the three model parameters are adjusted. In order to assess the quality of this model, we calculate the mean square error (MSE) obtained between all the experimental and predicted values of settlement. MSE is defined as 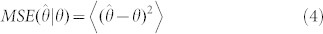
where 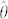 is the vector of predicted values for each settlement test and *θ* is the vector of the corresponding measured values. In Fig. 5b we have plotted the cumulative distribution function of MSE between 360 experimental tests: 40% of the settlement curves are predicted with an error below 5% and in 60% of tests the error is below 10%. This indicates that the model based on the Chicago fit provides a quite accurate estimation of the evolution of settlement in coarse granular materials and, what is more, the model parameters have a clear correlation with the packing fraction and loading parameters and can be obtained by independent tests.

## Discussion

In this work, we carried out a parametric study on a full-scale ballast track in order to test various models for the prediction of settlement in coarse granular materials under cyclic forcing. The settlement as a function of the number of cycles occurs in three stages with nearly the same functional dependence but varying with the initial state of the material and vibration amplitude. We showed that a model based on the Chicago density relaxation law provides an accurate estimation of settlement for a high number of cycles with parameters that reflect the loading parameters and initial mechanical state of the material and can be measured by independent tests. This model, together with its testing procedure, provides a reliable approach in applications where long-term settlement needs to be estimated in coarse granular materials.

Our findings raise also a fundamental question as to the predictability of the mechanical response of granular materials despite their natural variability and complex shapes of ballast grains. The behaviour is chaotic in the sense that small variations in the initial configuration of a granular packing are expected to be amplified with the number of loading cycles. This is not what we observe although fluctuations are observed between independent tests as shown in Fig. 3. This indicates that the fluctuating parts of consecutive incremental settlements average out and the system tends to a well-defined ultimate mechanical state. In other words, the mechanical state of the granular material under vibrations is determined by its distance from the ultimate state. This is consistent with the form of equation (1) where the relevant settlement parameter appears to be *τ_N_*/*τ*_∞_ with *τ*_∞_ only depending on the dimensionless acceleration parameter Γ. This feature of compaction can be generic to all systems characterized by jamming transition and in which a maximally jammed state may be defined^19^,^20^. Paradoxically, in such systems the long-term behaviour may be more accurately predicted than the short-term behaviour.

## Methods

The track model is composed of two monoblock sleepers lying on a ballast layer of thickness 0.35 m. The ballast layer is confined in a wood box of dimensions 4.11 m by 1.5 m. In this track model, the subgrade is represented by a wooden plate. The sleepers are fixed together by a rail section. A hydraulic cylinder applies the sinusoidal loading on the track. With the aim of providing a uniform load distribution, a beam is fixed to a hydraulic cylinder applying the overload on the rails. To study the influence of loading on settlement, we apply a sinusoidal load during 10^4^ cycles. The peak load is varied from 194 to 272 × 10^3^ N and the frequency from 3.3 Hz to 6 Hz. The cyclic loading is applied by a hydraulic cylinder on two sleepers placed on the granular bed. Before applying the cyclic loading, we characterized with several light penetration tests the initial mechanical state of the granular bed in our full-scale ballast track. In the course of cyclic loading, we measured with 4 non-contact optical sensors the evolution of settlement as a function of the number of cycles. The tests were carried out with 15 different combinations of the mean load and frequency and 6 independent initial conditions.

## Author Contributions

J.C.Q. performed the experiments and prepared the figures. J.C.Q., G.S., P.B. and F.R. worked on data analysis, did the theoretical work and reviewed the manuscript.

## Figures and Tables

**Table 1:** Cross-correlation coefficients between input and control parameters

	Γ	*q_d_*/*p*	*τ*_∞_/*H*_0_	*B*	*N*_0_	*H*_0_
Γ	1	0.03	0.91	−0.67	0.03	0.06
*q_d_*/*p*		1	0.02	−0.07	0.98	0.02
*τ*_∞_/*H*_0_			1	−0.64	0.01	0.04
*B*				1	−0.07	−0.32
*N*_0_					1	0.03
*H*_0_						1
